# Building health research systems: WHO is generating global perspectives, and who’s celebrating national successes?

**DOI:** 10.1186/s12961-016-0160-x

**Published:** 2016-12-28

**Authors:** Stephen R. Hanney, Miguel A. González-Block

**Affiliations:** 1 Health Economics Research Group, Institute of Environment, Health and Societies, Brunel University London, Kingston Lane, Uxbridge, UB8 3PH UK; 2 Universidad Anáhuac, Av. Universidad Anáhuac 46, Lomas Anáhuac, 52786, Huixquilucan, Mexico City Mexico

**Keywords:** National Institute for Health Research, Health research systems, WHO’s Global Observatory, Research funding, Priority setting, Capacity building, Public and patient involvement, Equity, Research networks, Research impact, Research waste

## Abstract

In 2016, England’s National Institute for Health Research (NIHR) celebrated its tenth anniversary as an innovative national health research system with a focus on meeting patients’ needs. This provides a good opportunity to reflect on how the creation of the NIHR has greatly enhanced important work, started in 1991, to develop a health research system in England that is embedded in the National Health Service.

In 2004, WHO identified a range of functions that a national health research system should undertake to improve the health of populations. *Health Research Policy and Systems* (HRPS) has taken particular interest in the pioneering developments in the English health research system, where the comprehensive approach has covered most, if not all, of the functions identified by WHO. Furthermore, several significant recent developments in thinking about health research are relevant for the NIHR and have informed accounts of its achievements. These include recognition of the need to combat waste in health research, which had been identified as a global problem in successive papers in the *Lancet*, and an increasing emphasis on demonstrating impact. Here, pioneering evaluation of United Kingdom research, conducted through the impact case studies of the Research Excellence Framework, is particularly important. Analyses informed by these and other approaches identified many aspects of NIHR’s progress in combating waste, building and sustaining research capacity, creating centres of research excellence linked to leading healthcare institutions, developing research networks, involving patients and others in identifying research needs, and producing and adopting research findings that are improving health outcomes.

The NIHR’s overall success, and an analysis of the remaining problems, might have lessons for other systems, notwithstanding important advances in many countries, as described in papers in HRPS and elsewhere. WHO’s recently established Global Observatory for Health Research and Development provides an opportunity to promote some of these lessons. To inform its work, the Observatory is sponsoring a thematic series of papers in HRPS focusing on health research issues such as funding flows, priority setting, capacity building, utilisation and equity. While important papers on these have been published, this series is still open to new submissions.

## Editorial

In 2016, England’s National Institute for Health Research (NIHR) celebrated its tenth anniversary as an innovative national health research system with a focus on meeting patients’ needs. This provides a good opportunity to reflect on how the creation of the NIHR has greatly enhanced important work, started in 1991, to develop a health research system in England that is embedded in the National Health Service (NHS) [1].

In 2004, WHO identified a range of functions that a national health research system should undertake in order to improve the health of populations [2]. The WHO framework for health research systems proposed that the two complementary intrinsic goals were “*the advancement of scientific knowledge and the utilization of knowledge to improve health and health equity*” ([2], p. 216). The framework included four key functions of a health research system and the associated operational components. First, stewardship, which includes defining and articulating a vision for a national health research system, identifying appropriate health research priorities and coordinating adherence to them, setting ethical standards for health research, and monitoring and evaluating the system. Second, financing, including securing research funds and allocating them accountably. Third, creating and sustaining the human and physical capacity to conduct, absorb and utilise health research. Fourth, producing and using research, including communicating it to inform health policy, strategies, practices and public opinion, and promoting it to develop new tools (drugs, vaccines, devices and other applications) to improve health [2].

*Health Research Policy and Systems* (HRPS) has taken particular interest in the pioneering developments in the health research system in England, where the comprehensive approach has covered most, if not all, of the items identified by WHO. The WHO framework recognises that, of course, there will be different configurations of the organisational structures in each country, with different bodies taking the lead in relation to different functions. While the NIHR was not explicitly aiming to apply the WHO framework at its outset, and does not have responsibility for all the functions in the United Kingdom, the framework nevertheless provides a useful approach for analysing the NIHR’s achievements, particularly in meeting the needs of the healthcare system.

To attempt such an analysis fully would be beyond the scope of an editorial. Nevertheless, there is considerable evidence on which to draw to illustrate the progress made. This evidence includes various papers in HRPS, and also the way in which significant developments in thinking about health research have been drawn upon in 2016 to examine the achievements of the NIHR. These developments are described more fully below, but they include the focus on combatting waste in health research, a problem highlighted in successive papers in the *Lancet* by Chalmers, Glasziou and colleagues [3–5], and the importance of demonstrating the impact of research. Here, the pioneering evaluation of United Kingdom research, conducted through the impact case studies of the Research Excellence Framework (widely known as the REF), has been important [6, 7].

In 2010, a paper in HRPS set out the considerable progress made in reforming the United Kingdom’s health research system. From 1991 onwards, new approaches were used to identify the priority needs of stakeholders working in the health system, and commissioning research to attempt to address those needs [1]. In particular, the Health Technology Assessment programme became increasingly successful in involving parts of the healthcare system in setting the research agenda on the treatments, drugs and devices on which to conduct research. The paper said of the Health Technology Assessment programme: “*its research is much used by various policymaking bodies*” whose role as receptor bodies enhances “*the status of the knowledge production involved in this type of research, which is important if its impact is to be sustained*” ([1], p. 12).

However, the 2010 paper also documented that a range of increasing problems had faced the health research system, including an apparent decline in the attractiveness of clinical academic medicine as a career. Thus, in 2005, Prof Dame Sally Davies, who had recently been appointed as Director of Health Research and Development in the Department of Health, set out comprehensive reform plans in *Best Research for Best Health* [8]. Following consultation, these plans led to the creation of the NIHR in 2006. The overall aim of embedding the research system into the NHS was promoted with the following mission: “*We aim to create a health research system in which the NHS supports outstanding individuals, working in world-class facilities, conducting leading-edge research, focused on the needs of patients and the public*” ([9], p. 5).

In the following 10 years, there have been many initiatives to address these points. Even by 2010 progress included an enhanced status for leading medical academics who could apply to become faculty in the College of NIHR Senior Investigators and whose funding was separate from the NHS’s patient care budget, the creation of Biomedical Research Centres and Units that were well-funded centres of research excellence and were co-located with leading medical facilities, and the expansion of the clinical research networks that provided an infrastructure supporting the conduct of clinical trials in all fields and across the country [1]; action has continued in these and other areas. This has turned into reality a claim made by Davies in the postscript to the 2006 document setting out the new strategy: “*we want to emphasise that the strategy does not consist simply of one or two ‘big ideas’ in isolation. We have to achieve a range of objectives which, although related, are individually quite distinct*” ([9], p. 36).

Various recent papers in HRPS can be used to provide illustrations of the many ways in which the NIHR has been successfully striving to fulfil its mission. Among them, an examination of ethics approval systems in various countries, which was generally positive about reforms in the system in England [10]; an account of how the NIHR has responded to the research needs of local government in the United Kingdom after responsibility for public health was transferred to it [11]; and an analysis of how an NIHR initiative to integrate research into the local healthcare system was successfully implemented in North West London [12].

The achievements of the NIHR in its first 10 years have been marked in various ways during 2016. In a blog on the NIHR’s site, Westmore [13] set out how the NIHR responded to Chalmers and Glasziou’s challenging estimate in 2009 [3], namely that 85% of all biomedical research is avoidably wasted because too much of it asks the wrong questions, is badly designed, not published or poorly reported. NIHR established the Adding Value in Research Framework to address such issues. Westmore listed actions in the NIHR which “*add value in research in many ways across the system*” and suggested that “*many of these elements aren’t commonplace in other health research systems around the world – doing them all could well be unique and we have been independently assessed as leaders in this area*” [13]. This independent support comes in the form of a further article in the *Lancet* [5] examining the response to Chalmers and Glasziou’s 2009 article.

We noted in an earlier editorial [14] that the REF in the United Kingdom provides a significant body of evidence demonstrating the impact of health research on health policies, practice and outcomes. An analysis of the database of REF case studies in the health research field identified a wide range of impact that came from diverse streams of NIHR support [15]. This work was used by PRiSM, the Policy Research in Science and Medicine unit from RAND Europe and the Policy Institute at King’s College London, as part of the evidence of the impact of the NIHR that they included in their report to mark its tenth anniversary [16]. This report described 100 examples of positive change and impact resulting from NIHR’s support for research. They were brought together under the caption “*NIHR at 10: 100 examples, 10 themes, 1 transformation*” ([16], p. 19), and highlighted in an accessible blog from the Policy Institute at King’s [17]. From the perspective of HRPS, it is significant to note the emphasis given to the NIHR being one system with a range of components. The ten themes of positive change and impact included key areas such as:

- “*putting patients and the public at the heart of all stages of research*”, which links to the mission of the NIHR;
- “*supporting, training and developing a diverse workforce in the NHS and academia*”, which links to capacity building efforts and embedding the research system within the NHS; and
- “*making the nation’s health and care system the best it can be*”, which links to the focus of meeting the needs of the NHS ([16], p. 2).

Despite all the successes, the NIHR is also aware of various areas where performance should be improved, especially in the context of a further study in HRPS that highlighted the desirability of reducing the time for research findings to be developed and implemented [18]. NIHR set up the Push the Pace project, which is now in its second phase [19]. It is addressing issues such as reducing the delays caused by the NIHR contracting system, improving dissemination and ensuring that the input from evidence users “*is used effectively to improve commissioning of research to better meet NHS/wider public health need*” [19].

The above discussion about the NIHR suggests many issues that might be relevant for health research systems worldwide, including the sustained attempt to embed the health research system widely into the healthcare system, the careful and inclusive priority setting involving many groups, especially patients and the public, the wide-ranging efforts to identify training and support needs in order to build and sustain research capacity, and the need for continual improvements to address problem areas and streamline the bureaucracy.

Of course, a great deal of innovative and important work is already going on in many countries in relation to these and other issues, and such initiatives are often captured in papers published in HRPS and elsewhere. These, too, could help inform the analysis of health research systems more widely. For example, Kirigia et al. [2] drew on the WHO framework and other sources to develop an African national health research systems barometer to monitor performance and “*guide policymakers to locate sources of poor performance and to design interventions to address them*” ([20], p. 1). The WHO framework was also used to inform a regional collaborative approach to health research development in the West African Health Authority and, according to Aidam and Sombié, the “*improved research partnerships and funding helped strengthen local health research environments*” ([21], p. 1).

In addition to systems level analysis, HRPS has also published recent papers on specific elements of health research systems. The role of networks is increasingly seen as important, and Fonseca and Zicker [22] applied social network analysis to produce a 20-year (1995–2014) retrospective longitudinal evaluation of Brazilian dengue research networks that provides relevant information for research policy and planning. Both Uzochukwu et al. [23] and Makkar et al. [24] importantly looked at the context within which health research is used. The former analysed policymakers’ and researchers’ capacity assets, needs and perspectives in southeast Nigeria [23]. The latter developed a way to measure an organisation’s capacity to use research in policymaking, with pilot-testing in Australia [24]. Wider social trends are also important with authors in HRPS papers rightly giving increasing attention to issues such as gender in health research systems in the United Kingdom and elsewhere [25–27].

Some, if not necessarily all, of the lessons from the NIHR experience are ones that WHO’s newly established Global Observatory for Health Research and Development might be able to promote. Furthermore, the Global Observatory is funding a series of papers in HRPS to inform its work [28]. Papers published so far have covered various key aspects of health research systems. For example, Cole et al. reviewed Malawi’s Health Research Capacity Strengthening Initiative, which was a national systems-strengthening programme that involved “*national priority setting, decision-making on funding, and health research actor mobilization*” ([29], p. 1). Woodward et al. [30] highlighted the importance of agreeing priority areas for the research funding made available from donors’ investments in the health sectors of fragile and conflict-affected states. They described an 18-month process to develop a consultative research agenda and questions for health systems research in which a wide range of stakeholders participated and which produced a useful starting point. Gotham et al. [31] examined the landscape of current policies and practices in relation to global health equity in United Kingdom universities’ research, and suggested various improvements.

The Global Observatory is particularly focused on the analysis of flows of research funding and three papers cover aspects of this. Through comparing total publications and citations with research investment to United Kingdom institutions in HIV, tuberculosis and malaria, Head et al. [32] were able to provide new evidence to inform research investment strategies for policymakers, funders, academic institutions and healthcare organisations. Viergever and Hendriks [33] described the progress they are making in the highly important tasks of increasing transparency about who the main funders of health research are globally, what they fund and how they decide on what gets funded, and improving the evidence base for various funding models.

Finally, Carter et al. [34], from the United Kingdom’s Medical Research Council, described how the various funding organisations in the United Kingdom health research system worked together to develop and apply the Health Research Classification System, which provides a consistent approach for comparing expenditure on health research. Its use “*has provided benefit both to individual participatory funders and in coordinating initiatives at a national level….The United Kingdom approach to landscaping analyses could be readily adapted to suit other groups or nations, and global availability of research funding data would support better national and international coordination of health research*” ([34], p. 1). This type of approach is fundamental to what the Global Observatory is attempting to achieve. It also illustrates how the various funding organisations within the English health research system are increasingly collaborating while maintaining their distinct roles.

Further papers have been submitted to the WHO Global Observatory’s Thematic Series in HRPS and are under review. They include ones related to exercises by development agencies in the United Kingdom and Canada related to priorities for health research investment, capacity building, use of research and equity. Another paper draws on the findings from the reviews of studies assessing the impact of health research programmes. From these, it is able to identify factors in the organisation of health research programmes that might be linked to achieving impact on policies, practice and health outcomes. However, the call for the WHO series is still open and Adam et al. [28] set out the full range of topics on which the Global Observatory will sponsor papers.

The experience and lessons learnt in the NIHR, as described above, and the papers on health research systems elsewhere, also provide examples of a range of topics that might be relevant to inform the work of the Global Observatory, and would thus be considered for the series. Additionally, papers are particularly welcome from countries and regions not so far represented in the series.

Finally, we return to the many successes of the NIHR and what we have previously called the Davies reforms [35]. The widespread domestic – and international – appreciation of what has been achieved by the NIHR emphasises just how worthwhile it is to strive to embed effective health research systems into healthcare systems in order to maximise the health of populations. It is appropriate to endorse the words of the Secretary of State for Health in England, Jeremy Hunt, who, at the event to celebrate the tenth anniversary of the NIHR, publically thanked Dame Sally Davies for her inspirational leadership during these 10 years.

## Abbreviations

HRPS: Health Research Policy and Systems
NHS: National Health Service
NIHR: National Institute for Health Research
REF: Research Excellence Framework
